# Systematic analysis of the antibacterial mechanisms of reuterin using the *E. coli* Keio collection

**DOI:** 10.1128/mbio.01432-25

**Published:** 2025-07-03

**Authors:** Jinyan Li, Teng Wang, Xi Yang, Yuhan Duan, Hirotada Mori

**Affiliations:** 1 State Key Laboratory of Swine and Poultry Breeding Industry, Guangdong Provincial Key Laboratory of Animal Breeding and Nutrition, Institute of Animal Science, Guangdong Academy of Agricultural Sciences, Guangzhou, Guangdong, China; 2 State Key Laboratory of Quantitative Synthetic Biology, Shenzhen Institute of Synthetic Biology, Shenzhen Institutes of Advanced Technology, Chinese Academy of Sciences, Shenzhen, Guangdong, China; 3 Faculty of Engineering, Shinshu University, Nagano, Japan; University of Würzburg, Würzburg, Germany

**Keywords:** reuterin, *Limosilactobacillus reuteri*, *E. coli*, Keio collection, high-throughput screening, redox balance, glutathione, antibacterial mechanism, biofilm

## Abstract

Reuterin is a broad-spectrum antimicrobial small molecule produced from glycerol by *Limosilactobacillus reuteri* through a one-step enzymatic reaction. In this study, we conducted a systematic analysis of the *Escherichia coli* Keio single-gene knockout mutant library to elucidate the molecular and cellular mechanisms of reuterin. Using sublethal concentrations of reuterin, we identified 159 sensitive mutants and 117 resistant mutants. GO and KEGG pathway analyses revealed that gene deletions in pathways related to the synthesis of aromatic amino acids, sulfur metabolism, and glutathione metabolism result in metabolic vulnerabilities that contribute to the antimicrobial mechanism of reuterin. This suggests that reuterin disrupts the intracellular redox balance, leading to reactive oxygen species production that the cells cannot manage, ultimately inducing cell death. Furthermore, the deletion of biofilm-related genes (*omrA* and *aaeR*) was found to increase biofilm formation, which functions as a structural barrier, reducing the uptake of environmental drugs into the cells and contributing to resistance against reuterin. Protein-protein interaction network analysis identified a series of antioxidant defense-related proteins that form the functional network underlying *E. coli*’s resistance to reuterin. Taken together, these findings enhance our understanding of the antibacterial activity mechanism of reuterin and provide potential targets and theoretical support for the development of new antimicrobial agents and further study of bacterial resistance mechanisms.

**IMPORTANCE:**

Reuterin is a low-molecular-weight compound with broad-spectrum antimicrobial activity. A systematic analysis was conducted using a library of non-essential gene deletion strains of *Escherichia coli* to elucidate the overall picture of its mechanism of action. While the generation of reactive oxygen species was suggested, this study was able to clarify the reactions occurring within *E. coli* cells that took up reuterin. It was revealed that pathways related to the synthesis of aromatic amino acids, sulfur metabolism, and glutathione metabolism play crucial roles in the antimicrobial mechanism. These findings not only deepen the understanding of the mechanism behind reuterin’s antimicrobial activity but also provide important information for considering the physiological significance of the antimicrobial activity of molecules secreted by other microorganisms in the intestinal environment.

## INTRODUCTION

Antibiotic resistance is a global challenge; therefore, there is an urgent need for new strategies to combat bacterial infections. Natural products with antibiotic-like properties, especially probiotics, have garnered significant attention due to their safety, unique mechanisms of action, and potential to inhibit pathogenic microorganisms. Among these, reuterin is a prominent small-molecule antimicrobial agent produced from glycerol by *Limosilactobacillus reuteri*.

Reuterin exhibits broad-spectrum antimicrobial activity, effectively inhibiting a range of gram-positive and gram-negative bacteria, as well as yeasts and fungi, reviewed by Sun et al. (1). Analysis of metabolites in the gastrointestinal tract of gnotobiotic mice revealed that *L. reuteri* can produce reuterin in the presence of glycerol; this finding supports the notion that reuterin may contribute to the probiotic function of *L. reuteri* within the gut (2).

In addition to antibacterial activity, many studies of reuterin focused on its regulatory mechanisms in complex environments. Recent studies found that it may regulate its production through quorum sensing, and others show that production of reuterin is influenced by cell density, suggesting that *L. reuteri* may regulate production by sensing cell density to avoid toxicity associated with high concentrations (3, 4). Quorum sensing, an important regulatory mechanism, allows bacteria to coordinate gene expression and metabolic activity by sensing population density, thereby enabling adaptive advantages (5). Although it is not clear yet whether reuterin functions as a signal molecule of quorum sensing for *L. reuteri*, current research indicates that it may influence group behavior and antimicrobial characteristics through interactions/signaling with other bacteria, suggesting that it could play a significant role in regulating microbial ecological balance (3, 4).

Reuterin is a complex mixture of molecules comprising primarily 3-hydroxypropanal (3-HPA), along with its spontaneously formed dimer, hydrate, and acrolein. Studies often refer to reuterin as 3-HPA, as this is the predominant antimicrobial component of the system; however, the antimicrobial activity of the reuterin system likely arises from the combined effects of 3-HPA and its conversion products, as these components exist in a dynamic equilibrium and contribute jointly to overall bioactivity (1, 6).

Reuterin may inhibit bacterial growth by inducing oxidative stress, damaging cell membranes, and causing DNA damage. Schaefer et al. (7) found that the interaction of reuterin with mercaptan groups within proteins and small molecules may cause cellular oxidative stress, thereby inhibiting the growth of bacteria. Engevik et al. (8) found that reuterin caused loss of membrane integrity in *Clostridioides difficile* and observed a 7.6-fold increase in DNA damage in a TUNEL assay, suggesting that reuterin affects bacterial cells by disrupting the cell membrane and causing DNA damage. Furthermore, molecular docking simulation studies conducted by Purnawita et al. showed that the active component of reuterin, 3-HPA, inhibits the activity of antioxidant enzymes such as the catalase family, further increasing intracellular reactive oxygen species (ROS) levels and inducing oxidative stress (9).

Although some studies, e.g., Schaefer et al. (7), explored the role of individual genes, such as *oxyR*, in the antibacterial mechanism of reuterin, most previous research relied on the detection of chemical substances and evaluation of changes in cellular characteristics, from which a mechanism was inferred. While these studies provide valuable insights, they lack systematic exploration at the genetic regulatory level, particularly with respect to understanding how genes regulate bacterial sensitivity or resistance to reuterin.

Here, we conducted a genome-wide systematic exploration of the mechanism underlying the activity of reuterin. To do this, we used the *Escherichia coli* Keio single-gene knockout mutant library, commonly known as the Keio collection (10), to examine the effects of loss-of-function. This library, which contains nearly 4,000 single-gene knockout mutants, is a valuable resource for high-throughput screening of *E. coli* mutants that are sensitive or resistant to reuterin. We adopted a systematic screening approach to identify key genes related to reuterin sensitivity and resistance and conducted bioinformatics analysis to uncover potential regulatory mechanisms. The findings provide important insight into the antibacterial mechanism of reuterin, not only deepening our understanding of its mode of action but also offering a theoretical basis for the development of antibacterial strategies targeting metabolic pathways or resistance. Additionally, this work lays the foundation for further elucidation of the molecular regulatory networks involved in bacterial responses to reuterin.

## RESULTS

### MIC of reuterin for *E. coli* K-12

Reuterin was prepared from 1 L of *L. reuteri* culture, which had a wet weight of 11.96 g; the concentration of the resulting reuterin solution was 28.81 mmol/L. In a liquid environment, the MIC of reuterin for *E. coli* BW25113 was 1.15 mM (Fig. S1).

### Evaluation of experimental reproducibility

To perform a systematic analysis of the antibiotic effects of reuterin on *E. coli*, we first determined the optimal parameters for growth profile analysis and then confirmed their reproducibility. The Colony-live system provides growth parameters such as conventional colony area (CONV), lag time of growth (LTG), maximum growth rate (MGR), and saturation point of growth (SPG), which are indicative of the growth status of each strain and are characterized by the Gompertz growth curve (11). The LTG parameter, which indicates the lag time before growth resumes after the stationary phase, is influenced significantly by the time spent by cells in a steady state, their genetic background, and factors that drive entry into the stationary phase (12) and reviewed by Bertrand (13). The CONV obtains the area of the entire colony, projected in two dimensions; however, the current transmission-type scanner cannot distinguish between structures such as polysaccharides on the cell surface of bacteria exhibiting a mucoid phenotype and the cells themselves.

Therefore, the MGR and SPG are more appropriate parameters for examining the effects of genetic background and environmental factors on cell growth. The Colony-live system requires most of the colonies growing to identify and quantify those colony growths. At concentrations over 1.0× and 2.0× the MIC, almost all *E. coli* cells cannot grow. Consequently, we examined the reproducibility of these parameters at reuterin concentrations of 0× the MIC and 0.5× the MIC.

A total of 3,903 nonessential gene deletion mutants, including some non-coding RNA (ncRNA) genes, were analyzed. Among the Keio collection, two strains (Δ*yeeJ* and Δ*gid*A) failed to grow on the cultivation plate, and two others (Δ*glm*Y and Δ*sra*A) exhibited significant variability in the repeat experiments, leading to their exclusion from subsequent analyses. All screening data are shown in Table S1.

**Fig 1:**
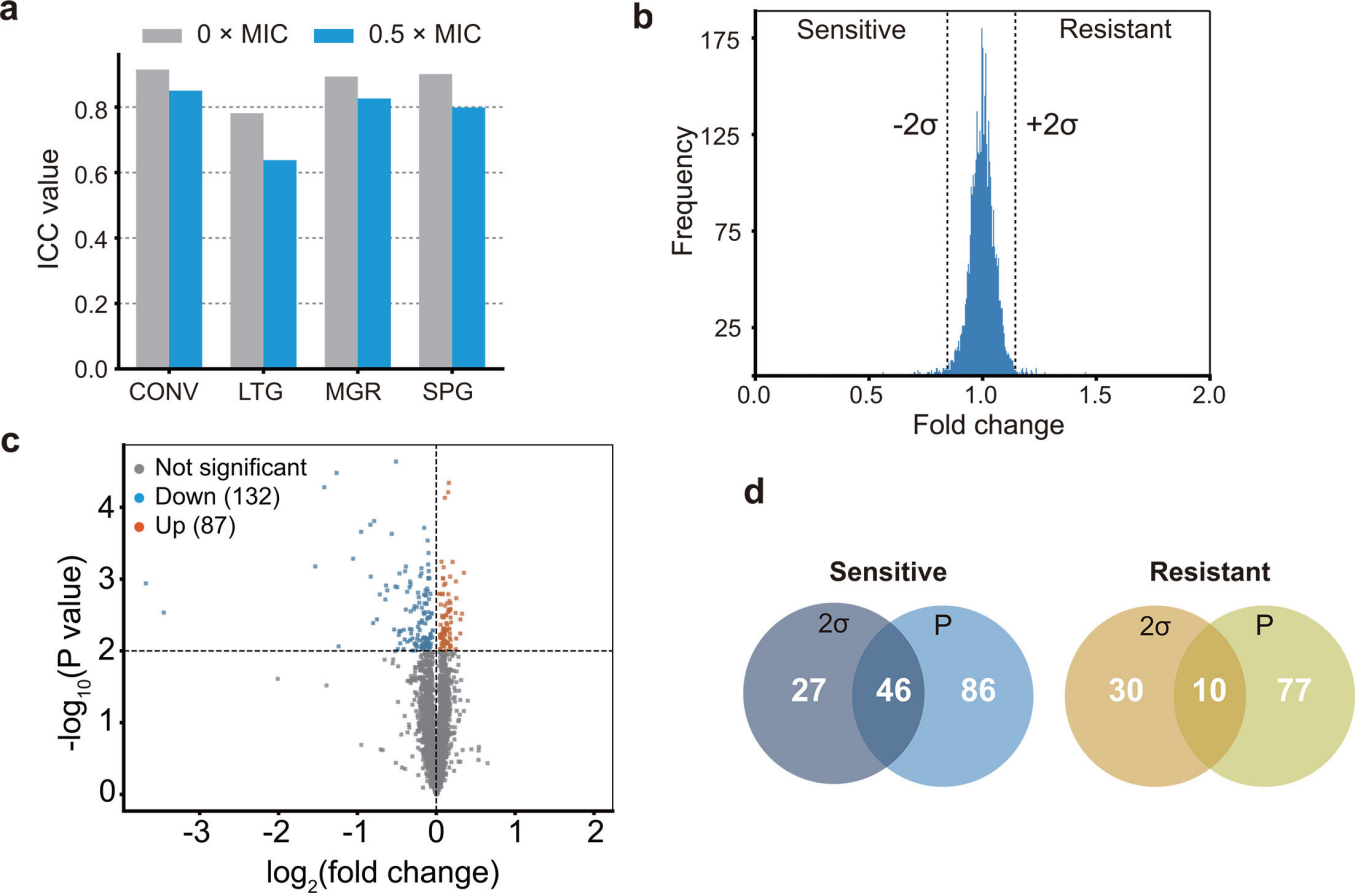
Analysis of reproducibility and screening of candidate genes. (**a**) Intraclass correlation coefficient values were calculated for different parameters under varying treatment conditions: 0.5× MIC reuterin in blue; 0× MIC (control) in gray. (**b**) Histogram depicting mutants screened using the ±2σ method, showing the distribution of standardized fold change values in the presence of reuterin. (**c**) Volcano plot of significant mutants, with fold change plotted on a log_2_ scale (*x*-axis) and *P* values plotted on a −log_10_ scale (*y*-axis). Statistically significant mutants (*P* < 0.01) showing sensitivity or resistance are shown in blue and orange, respectively. (**d**) Venn diagram illustrating the overlap of sensitive and resistant mutants identified by (2σ) the ±2σ method and (P) *P* value.

In the absence of reuterin, all parameters demonstrated high reproducibility (Fig. 1a), with intraclass correlation coefficient (ICC) values > 0.78. In the presence of 0.5× MIC reuterin, the ICC values for MGR (0.826) and SPG (0.799) were very consistent across four independent experiments, with MGR showing particularly high reproducibility. By contrast, the ICC value for LTG was relatively low, indicating greater variability among different strains under different treatment conditions.

Based on these results, we decided that MGR was the most reliable indicator for evaluating the effects of reuterin on bacterial growth.

### Comparison of different screening methods and identification of candidate mutants

As mentioned above, we utilized MGR to evaluate the antibiotic-like activity of reuterin against *E. coli*. We based our evaluation on fold changes in the MGR in the presence/absence of reuterin. First, we examined complete gene deletion strains and performed screening based on selection at a threshold of ±2σ and a statistical significance of *P* < 0.01 (based on a *t*-test; Fig. 1b and c). All genes selected according to these two criteria were used for further analysis. Figure 1d shows a Venn diagram illustrating the relation between the two selection methods by standard deviation (2σ) and *P*-value (*P*) as thresholds. As a result, we identified 159 reuterin-sensitive mutants and 117 resistant mutants, including ncRNA gene mutants. All of the screened candidates are listed in Table S2.

In the sensitive group, 46 genes were identified by both clusters classified by 2σ and *P*, indicating that these deletion mutants have a weakened ability to adapt with statistical significance under conditions of drug exposure. Additionally, although 27 genes had significant effects on growth, this was deemed to be weak statistical significance (*P* > 0.01). Another 86 genes did show statistical significance, although their contribution to biological function was considered less pronounced than that of the overlapping gene group. In the resistant group, 10 overlapping genes exhibited significant growth effects. Furthermore, 30 genes conferred extreme resistance but were considered to have low statistical significance. By contrast, 77 genes showed significant differences, but their resistance phenotypes were relatively mild. By comparing the screening results obtained using the two methods, we were able to simultaneously identify biologically extreme and statistically significant candidate genes.

The growth curves of three representative mutants from each of the sensitive and resistant groups are presented in Fig. S2. The growth curves of resistant mutants were lower than those of the control group; however, the fitted analysis revealed an increase in the MGR (fold change > 1). Resistant mutants exhibited a higher MGR in the presence of reuterin, suggesting early adaptation or a rapid response to the drug; however, continued exposure inhibited overall growth.

### COG and GO functional annotation and analysis

#### COG annotation analysis

After analyzing the results of the comprehensive reuterin-sensitivity test using the Keio collection of single-gene knockouts, we next conducted Clusters of Orthologous Genes (COG) and Gene Ontology (GO) analyses to identify similarities in the structure and function of the selected genes.

**Fig 2:**
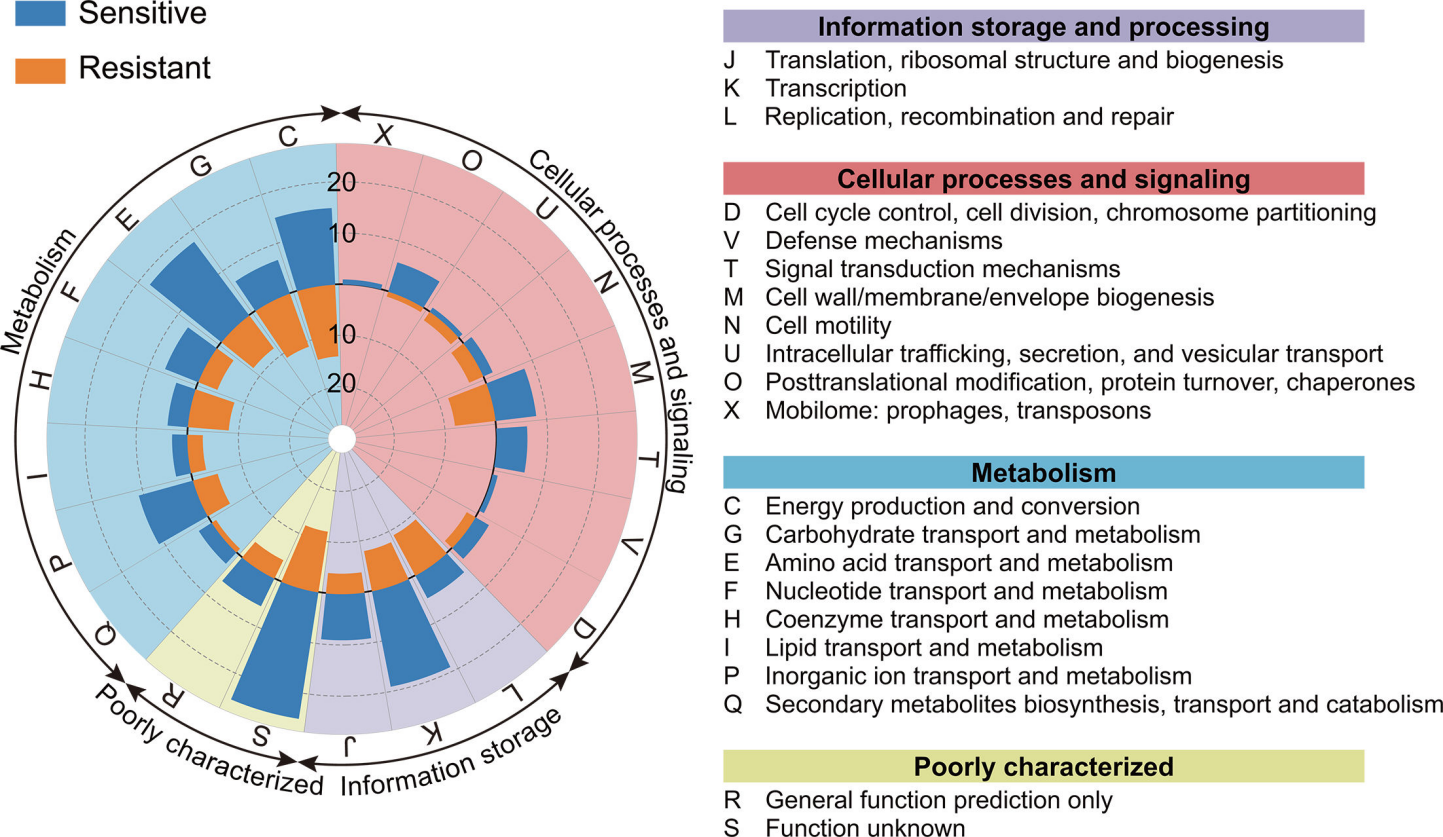
COG annotation analysis. The blue bars represent the sensitive group, and the orange bars represent the resistant group. The circular grid lines provide scale reference, and the length of the bar indicates the gene count distribution within each COG subcategory. Different colored sectors represent the four major COG functional classes. The table beside the figure lists detailed functional information for each COG subcategory.

Functional annotation of all genes in the sensitive and resistant groups (*n* = 260; 16 genes had no COG annotation) led us to classify them into four major categories: information storage and processing, cellular processes and signaling, metabolism, and poorly characterized (distributed across 21 subcategories; Fig. 2). We found that the gene count distribution within the subcategories varied between the sensitive and resistant groups.

Within the information storage and processing category, most of the sensitive genes were concentrated in the transcription (K, *n* = 19) and the translation, ribosomal structure, and biogenesis (J, *n* = 9) subcategories, whereas the resistant group had only *n* = 8 and *n* = 4 genes, respectively, in these subcategories. By contrast, more genes (*n* = 9) in the resistant group than in the sensitive group (*n* = 5) belonged to the replication, recombination, and repair (L) subcategory.

In the cellular processes and signaling category, genes related to signal transduction mechanisms (T, *n* = 6) were found only in the sensitive group, whereas genes related to cell wall/membrane/envelope biogenesis (M, *n* = 8) were distributed equally among both groups. Additionally, sensitive genes were more prevalent than resistant genes in the post-translational modification, protein turnover, and chaperones subcategory (O, *n* = 6 vs *n* = 1, respectively).

In the metabolism category, sensitive genes were distributed primarily in the amino acid transport and metabolism subcategory (E, *n* = 18). By contrast, more genes in the resistant group belonged to the coenzyme transport and metabolism (H, *n* = 8) and carbohydrate transport and metabolism (G, *n* = 11) subcategories. With respect to energy production and conversion (C), the two groups had similar gene counts (sensitive group *n* = 15, resistant group *n* = 14).

In the poorly characterized category, sensitive genes were more abundant than resistant genes in both the function unknown (S category, *n* = 25) and general function prediction only (R category, *n* = 6) subcategories (*n* = 12 and *n* = 4, respectively).

#### GO enrichment analysis

GO enrichment analysis revealed that the genes in the sensitive and resistant groups were spread differently across three groups: Biological Process (BP), Cellular Component (CC), and Molecular Function (MF). To obtain more information about functional trends, we used the EASE score, which is the enrichment score of a modified Fisher’s exact *t*-test *P* value (14, 15). We set the threshold for the sensitive group at EASE score <0.05, while for the resistant group, the thresholds were set at 0.1 for BP and MF, and 0.3 for CC. We then visualized and evaluated the enrichment analysis results based on the fold enrichment (FE) value and the (*P*) value (EASE score).

**Fig 3:**
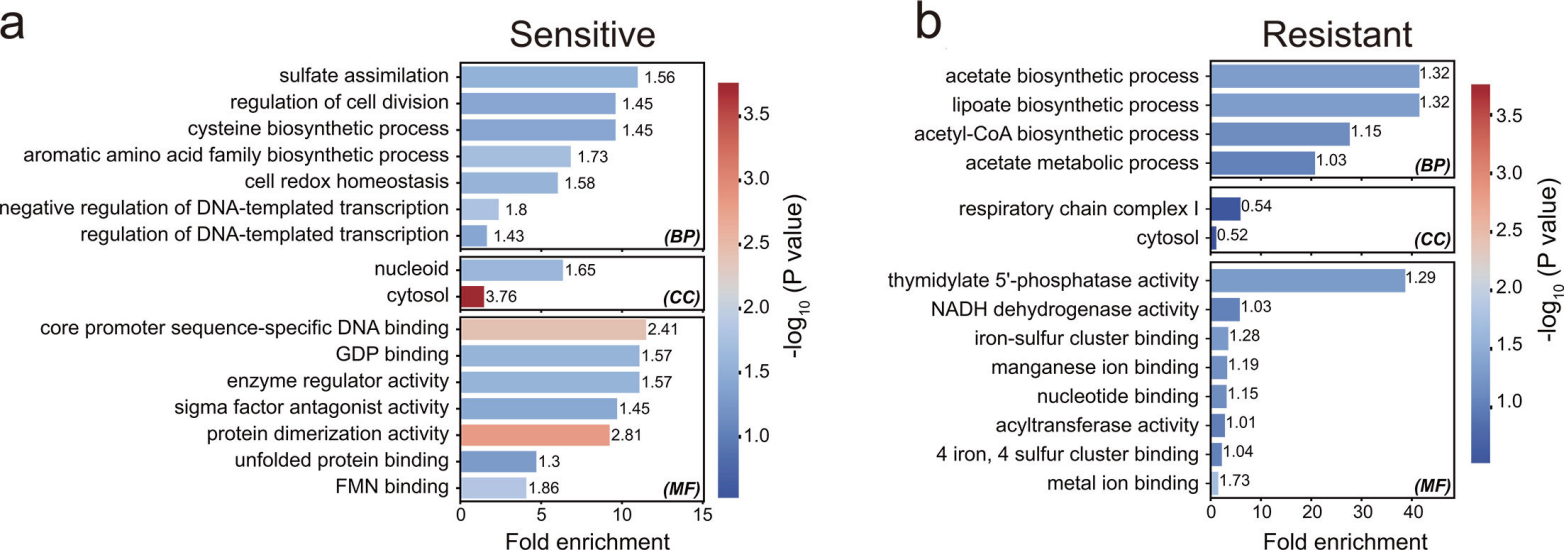
Functional enrichment analysis of candidate genes. (**a**) GO enrichment analysis of sensitive genes (*P* value [EASE] < 0.05 and count ≥ 2). (**b**) GO enrichment analysis of resistant genes, using more relaxed *P* value thresholds of 0.1 for BP and MF, and 0.3 for CC, and a count of ≥2, to capture additional biological information.

Sixteen categories were enriched in the sensitive group (Fig. 3a). The BP terms that increased most significantly were “sulfate assimilation” (FE = 10.96, *P* = 0.028) and “the aromatic amino acid family biosynthesis process” (FE = 6.82, *P* = 0.018). Additionally, “cellular redox homeostasis” (FE = 6.02, *P* = 0.026) also had a high enrichment value. “Cytosol” showed significant enrichment in the CC category (FE = 1.47, *P* = 0.0001). We found that “protein dimerization activity” (FE = 9.24, *P* = 0.001), “core promoter sequence-specific DNA binding” (FE = 11.50, *P* = 0.004), and “FMN binding” (FE = 4.08, *P* = 0.014) were the most important MF terms.

Genes in the resistant group showed an enrichment. The top enriched BP terms were “lipoate biosynthesis” (FE = 40.99, *P* = 0.0476) and “acetate biosynthesis” (FE = 40.99, *P* = 0.0476). “The respiratory chain complex I” (FE = 5.86, *P* = 0.2899) exhibited gene enrichment in the CC category. As shown in Fig. 3b, the most significant *P* value in the MF group was for “metal ion binding” (FE = 1.50, *P* = 0.0185), and the most significant FE value was for “thymidylate 5′-phosphatase activity” (FE = 38.22, *P* = 0.051).

### KEGG pathway analysis

#### KEGG enrichment analysis

COG and GO analyses describe the functional attributes and bias of the screened genes. To assess the functional distribution and synergistic effects of candidate genes within cellular pathways, we conducted Kyoto Encyclopedia of Genes and Genomes (KEGG) pathway analysis to elucidate the molecular mechanisms underlying changes in reuterin sensitivity induced by gene knockout.

**Fig 4:**
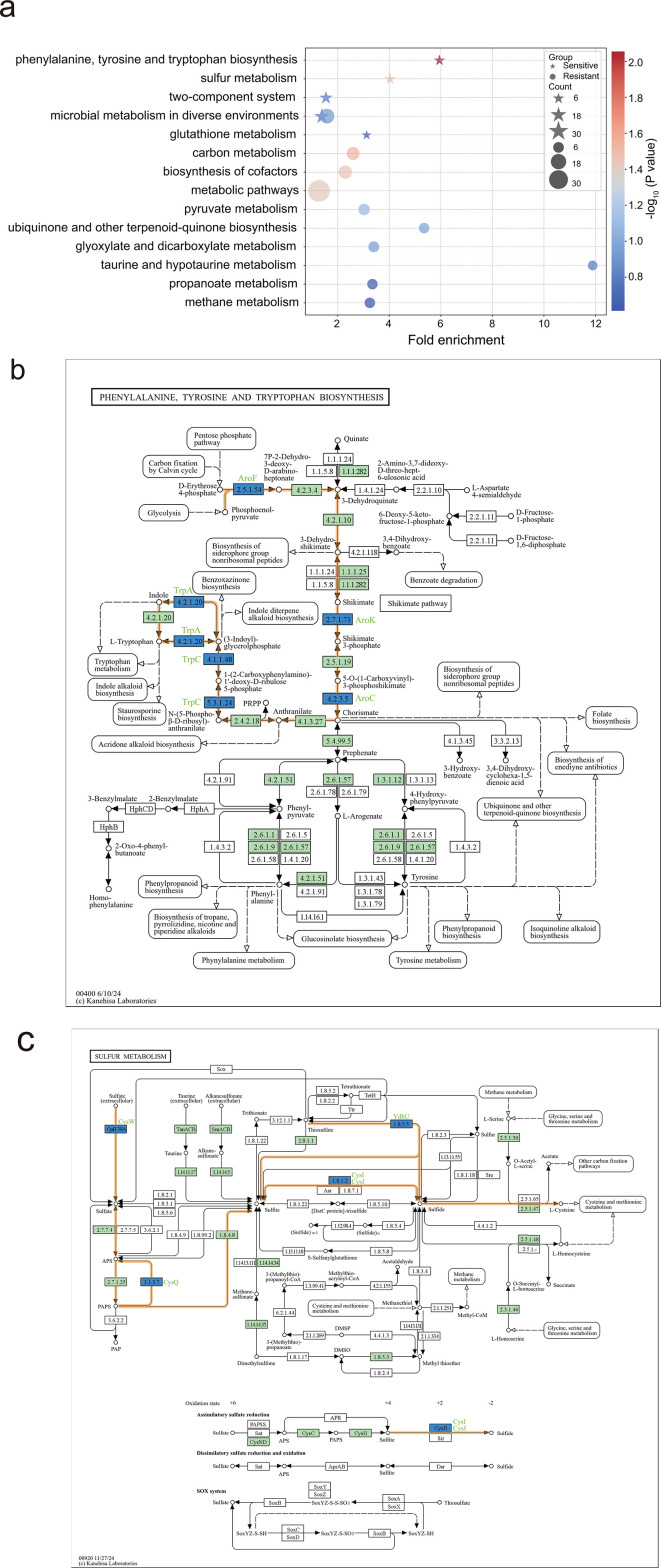
KEGG pathway analysis. (**a**) KEGG pathway enrichment bubble chart. The bubble color gradient represents −log_10_ (*P* value), and bubble size indicates the number of genes (count). Asterisks denote the sensitive group, and circles denote the resistant group (*P* value < 0.25 and count ≥ 2). (**b**) The phenylalanine, tyrosine, and tryptophan biosynthesis pathway. (**c**) The sulfur metabolism pathway. Green boxes denote *E. coli* proteins, blue boxes denote sensitive candidate gene products, and thick orange lines highlight the positions of candidate genes in the pathway.

First, the KEGG enrichment analysis results showed an enrichment trend in 14 pathways for both the sensitive and resistant groups (*P* value < 0.25 and gene count ≥ 2); these included 13 metabolic pathways and one environmental information processing pathway and involved 67 genes (Fig. 4a; Fig. S3). The significance of the enrichment pathways, as quantified by the *P* value and FE values, revealed clear differences between the sensitive and resistant groups; one exception was the shared “Microbial metabolism in diverse environments” pathway. The sensitive group showed enrichment primarily of the “biosynthesis of phenylalanine, tyrosine, and tryptophan” (FE = 5.95, *P* = 0.008); “sulfur metabolism” (FE = 4.03, *P* = 0.031); and “glutathione metabolism” (FE = 3.12, *P* = 0.244) pathways. By contrast, the resistant group showed enrichment mainly of “carbon metabolism” (FE = 2.59, *P* = 0.008), “cofactor biosynthesis” (FE = 2.29, *P* = 0.033), and “pyruvate metabolism” (FE = 3.02, *P* = 0.076).

#### KEGG pathway network analysis

Next, we mapped all candidate genes to pathways in the KEGG database. The KEGG database did not register 70 of the 276 candidate genes, but it did locate and involve 206 gene products in 87 pathways (Table S3). We observed that many candidate genes were either adjacent to each other or located in relatively close proximity within specific pathways, particularly in enriched pathways. For example, in the sensitive group, *aroCFK* and *trpAC* within the “biosynthesis of phenylalanine, tyrosine, and tryptophan” pathway and *cysIJ* and *ydhU* within the “sulfur metabolism” pathway (Fig. 4b and c) were identified as “molecular interaction” sites. In the figure, gene positions are marked by blue boxes, and pathways with tightly packed genes are highlighted by thick orange lines. These genes are considered to have a significant impact on the production of specific metabolites such as chorismate, sulfate, sulfite, and sulfides, all of which may be crucial for the biological activity of reuterin.

**Fig 5:**
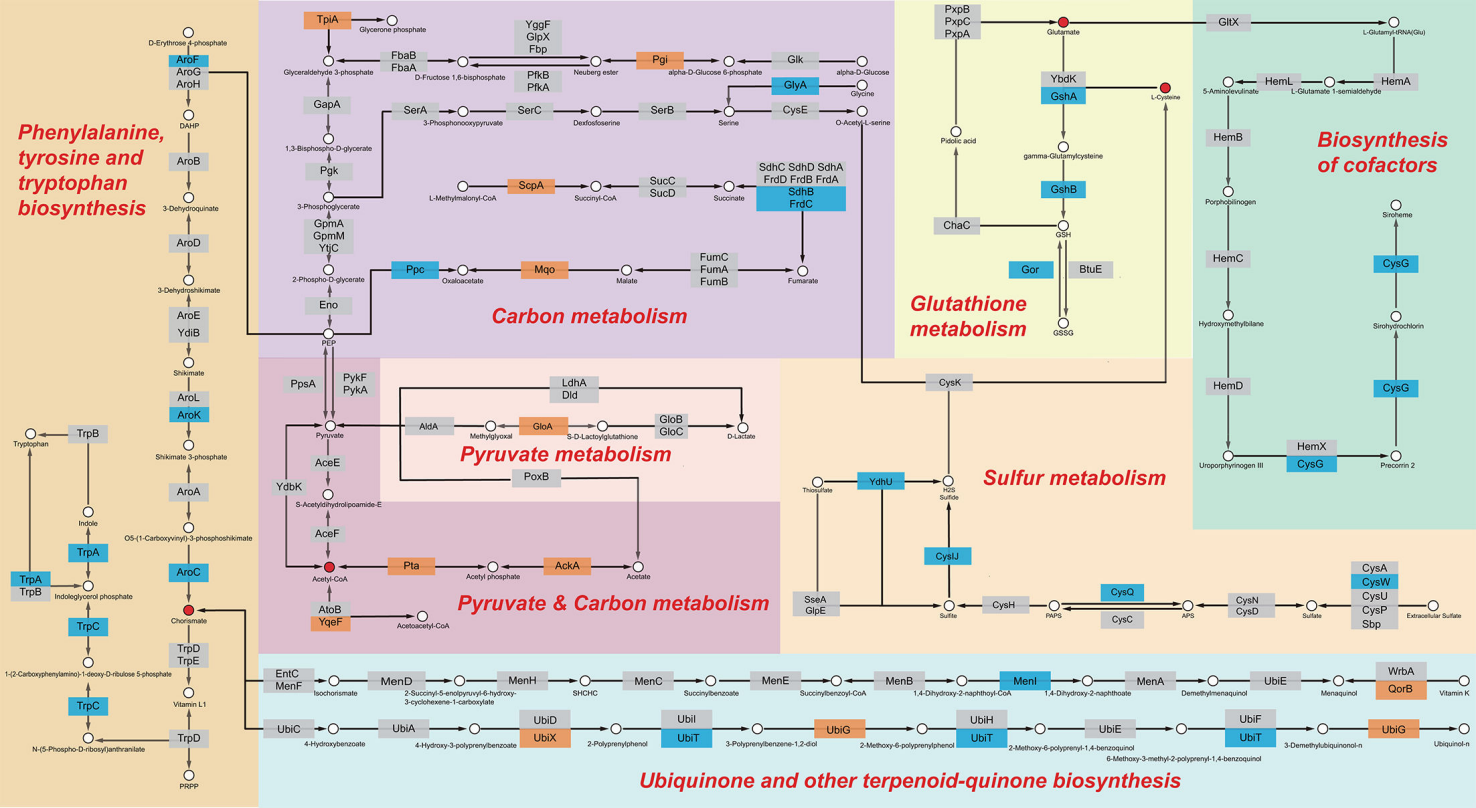
KEGG integrated metabolic network. The seven KEGG metabolic maps are connected and simplified through common metabolites such as L-cysteine, glutamate, and chorismate. The blue box indicates sensitivity due to gene deletion, while the orange box indicates resistance.

Common metabolites within actual cellular processes connect individual metabolic pathways in the KEGG database. To understand the overall cellular response induced by reuterin, we excluded pathways belonging to broader categories such as metabolic pathways and microbial metabolism in diverse environments. We then simplified pathways with dense gene distributions, while preserving metabolic intermediates. Finally, we connected pathways with shared metabolites, resulting in an integrated diagram. As shown in Fig. 5, the integrated network includes “biosynthesis of phenylalanine, tyrosine, and tryptophan,” “glutathione metabolism,” “sulfur metabolism,” “carbon metabolism,” “pyruvate metabolism,” and “cofactor synthesis,” as well as “biosynthesis of ubiquinone and other terpenoid quinones.” Chemicals such as L-cysteine, glutamate, chlorate, and others help these pathways connect by acting as metabolic bridges to keep the synthesis network intact. The blue boxes in the figure denote sensitive group genes that are involved mostly in the “biosynthesis of aromatic amino acids, sulfur, glutathione, and cofactors” pathway. Genes (orange) in the resistance group are involved in carbon metabolism, pyruvate metabolism, and cofactor biosynthesis.

### Protein-protein interaction network analysis

To identify more proteins that might play important roles in metabolic or signaling pathways (core nodes) from the perspective of protein-protein interaction (PPI), candidate gene data downloaded from the STRING database (16) were used for PPI network analysis by Cytoscape. The intersection of the top 10 proteins, ranked by four algorithms described in Materials and Methods, was used to identify proteins that may have important functions during the cellular response to reuterin.

**Fig 6:**
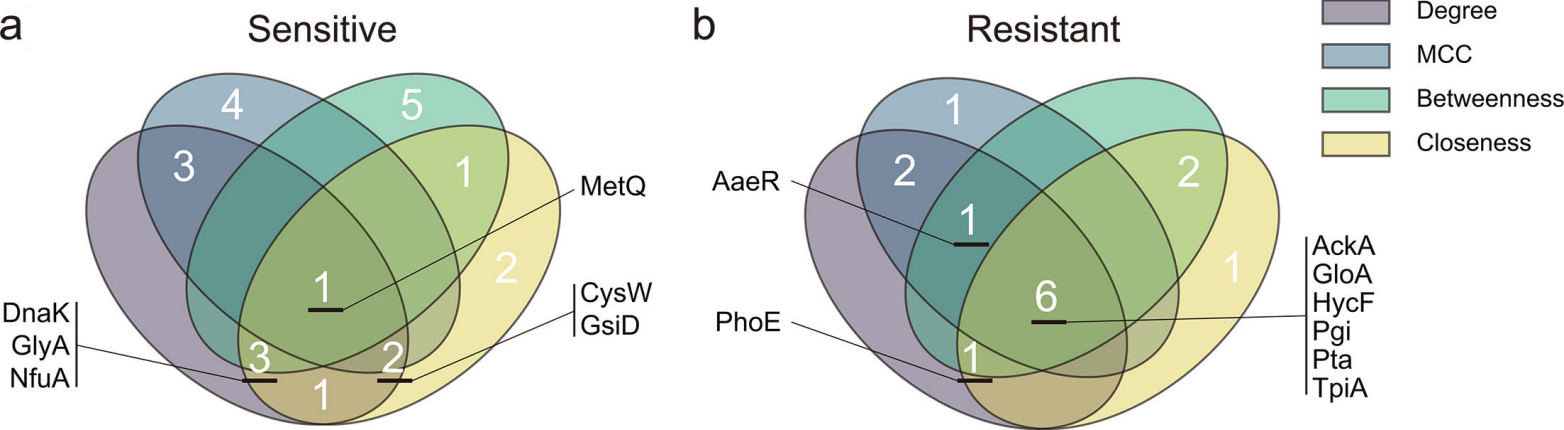
PPI network analysis. A Venn diagram of top 10 proteins in the sensitive and resistant groups (derived by the four algorithms). (**a**) Sensitive group. (**b**) Resistant group. Proteins were selected with medium confidence (0.4), FDR ≤ 0.05, and strength ≥ 0.01.

In the sensitive group (Fig. 6a), MetQ was the only protein that stood out in all of the algorithms. MetQ is an important substrate-binding protein required by the methionine ABC transport system and is closely linked to the metabolism and uptake of methionine and its derivatives (17). GsiD and CysW were identified by the Degree, Maximal Clique Centrality (MCC), and Closeness algorithms as membrane subunits of the glutathione (18) and sulfate (19) ABC transport systems, suggesting that metabolite transport may play a critical role in cellular responses to reuterin. Moreover, GlyA, NfuA, and DnaK were screened as core proteins by the Degree, Betweenness, and Closeness algorithms. These proteins are involved in key cellular processes such as one-carbon metabolism (20), iron-sulfur cluster assembly (21, 22), and protein folding (23), reflecting the importance of metabolic regulation and stress responses.

All four algorithms identified HycF, AckA, GloA, TpiA, Pta, and Pgi as core proteins in the resistant group PPI network (Fig. 6b). HycF is a component of the formate hydrogenlyase complex, specifically hydrogenase 3, and is a 4Fe-4S ferredoxin that participates in electron transfer (24). AckA and Pta, key enzymes in the acetate metabolism pathway, are responsible for the conversion of acetyl phosphate to acetate during energy metabolism (25, 26). GloA catalyzes the conversion of methylglyoxal to D-lactate and is an important enzyme for detoxification of methylglyoxal (27, 28). TpiA and Pgi are core enzymes in the “glycolysis and gluconeogenesis” pathways, regulating the balance of carbon metabolism (29, 30), which is crucial for maintaining cellular metabolic homeostasis. Additionally, AaeR was selected by the Degree, MCC, and Betweenness algorithms. It is a transcriptional regulator that controls the efflux of aromatic carboxylic acids by activating the *aaeXAB* operon and may also be involved in biofilm formation and regulation of virulence genes (31–33). PhoE, identified by the Degree, Betweenness, and Closeness algorithms, is a bacterial outer membrane porin (34), and its central position in the PPI network suggests a potential role in metabolic balance. Figure S4 shows a PPI network map based on Degree values; the positions of these proteins within the interaction network are shown.

## DISCUSSION

### Sensitivity analysis of mutants

Our genome-wide screening identified 276 genes modulating reuterin sensitivity. Sublethal reuterin screening identified mutants with disrupted redox-related pathways (e.g., sulfate assimilation and aromatic amino acid biosynthesis) as highly susceptible, while those defective in lipoate/acetate metabolism exhibited enhanced tolerance during the exponential growth phase. KEGG pathway analysis further highlighted enriched aromatic amino acid and sulfur metabolism in sensitive strains, and energy/cofactor biosynthesis pathways in resistant strains, implicating metabolic interference as a core antibacterial mechanism.

### Disturbance of the metabolic network

Construction of the core metabolic network further revealed the functional importance of key enzymes (Fig. 5). AroC, which encodes chorismate synthase, is a crucial enzyme required for the biosynthesis of aromatic amino acids, as it catalyzes the conversion of 5-enolpyruvylshikimate-3-phosphate to chorismate (35), which serves as a precursor for the biosynthesis of aromatic amino acids and cofactors (ubiquinone and other terpenoid-quinone) (36). Loss of AroC function may lead to a metabolic imbalance, increasing sensitivity to reuterin.

The GSH synthesis pathway is closely coupled with sulfur metabolism and cofactor biosynthesis through the shared metabolite L-cysteine. Loss of CysG impairs siroheme synthesis, which is an essential cofactor for the reduction of sulfite to sulfide (37), leading to reduced sulfide supply. This affects the levels of L-cysteine and GSH, weakening antioxidant defense and increasing metabolic fragility under oxidative stress (38). This is consistent with previous studies suggesting that reuterin induces oxidative stress (1).

The metabolic strategy in the resistant group is centered around carbon metabolism and pyruvate metabolism, with acetyl-CoA serving as a bridge between these two pathways. Deletion of AckA shows an increased level of acetyl-phosphate, whereas deletion of Pta reduces the level of acetyl-phosphate (39). However, deletion of both genes shows increased expression of the TCA cycle (40) and increased resistance to hydroxyurea, leading to depletion of the dNTP pool (39, 41). Additionally, the loss of GloA impairs the detoxification of methylglyoxal. Although its accumulation exacerbates redox imbalance, it can activate stress response pathways such as the *nemRA-gloA* operon (42), enhancing the mutant’s adaptability under reuterin treatment. This metabolic regulation and signaling response may be one of the key reasons underlying the resistant phenotype observed in these mutants.

### Role of biofilm barriers in resistance formation

Extraction of core functional proteins from the PPI network enabled us to elucidate metabolic regulation and stress response mechanisms that play a role in cellular responses to reuterin. Core proteins in the sensitive group, such as MetQ, CysW, and GsiD, are associated primarily with metabolite transport, sulfur metabolism, and antioxidant defense. Their deletion results in impaired transport of metabolites, including methionine, sulfate, and GSH, revealing vulnerabilities in metabolic regulation—particularly in metabolite transport and redox balance.

The results of core protein screening within the resistant group identified AckA, GloA, HycF, Pgi, Pta, and TpiA complementing and confirming the conclusions drawn from pathway analysis. This suggests that metabolic flux reprogramming and signal regulation play significant roles in the development of reuterin resistance by the mutants.

The deletion of AaeR is considered to reduce the extracellular efflux of aromatic carboxylic acids by repressing the activation of the *aaeXAB* operon, which may lead to an increase in the intracellular amounts of the same substances (32). Aromatic molecules have been reported to have ROS scavenging effects (43). Additionally, the deletion of AaeR leads to the induction of biofilm formation (33). Furthermore, PhoE, an outer membrane channel protein primarily involved in passive diffusion of anionic small molecules, was identified as a core protein in the resistant group (44). Its deletion may regulate the overall permeability of the outer membrane barrier, thereby enhancing the cell’s tolerance to environmental stress indirectly and enabling the mutant to exhibit a resistant phenotype. Moreover, the *yqcE* mutant, which is predicted to be a membrane transporter for glycerophosphodiesters, exhibited resistance to reuterin and may be involved in the initial molecular recognition of reuterin, though further experimental validation is required.

It is noteworthy that we also constructed knockout mutants of some of the ncRNA genes located in intergenic regions; these mutants are distinct from the protein-coding gene knockout library from the Keio collection. These knockout strains were also subjected to reuterin-sensitivity tests. The results revealed that knockout of *omrA*, a small regulatory RNA, caused significant resistance to reuterin. OmrA negatively regulates the mRNA levels of downstream genes such as *ompR* (45), *csgD* (46), and *dgcM* (47); it also downregulates the production of curli fibers, which are a major component of biofilms (48). Deletion of this gene may lead to increased production of curli fibers, enhanced biofilm biosynthesis, and reduced reuterin uptake by the cell, resulting in drug resistance; however, we must consider the overlapping regions of ncRNAs and the regulatory signaling regions of neighboring genes.

### Conclusion

In summary, based on the findings reported here, reuterin’s antibacterial mechanism likely involves two synergistic strategies: exploitation of metabolic vulnerability and structural barrier modulation. By inducing oxidative stress, reuterin amplifies defects in redox-sensitive pathways, such as aromatic amino acid, sulfur, and glutathione metabolism, leading to redox imbalance and metabolic instability. In wild-type bacteria, acetate metabolism genes, such as *ackA* and *pta,* contribute to sensitivity, whereas their deletion alleviates stress via metabolic flux rewiring. Structural adaptations further mediate resistance. Deletion of *phoE* may reduce membrane permeability, and deletions of *aaeR* and *omrA* enhance biofilm formation as a structural barrier, leading to limited drug transport. Additionally, reuterin-induced methylglyoxal accumulation may activate the *nemRA-gloA* operon, enhancing cellular antioxidant capacity. This study systematically maps reuterin’s cellular targets, linking metabolic fragility and biofilm dynamics to antibacterial activity. Further validation of biofilm and transport roles will deepen mechanistic insights.

## MATERIALS AND METHODS

### Bacterial strains

*L. reuteri* ATCC 55730 (SD2112) was obtained from ATCC. The *E. coli* Keio collection (10) and its host strain BW25113 (49) were used. Non-coding RNA gene deletion mutants were generated using λ RED recombination (W. Nomura, unpublished).

### Preparation of reuterin and measurement of the minimum inhibitory concentration for *E. coli* K-12 BW25113

Reuterin was synthesized from *L. reuteri* following Doleyres et al. (50) with modifications (see Supplemental methods), and the concentration was measured as described by Ortiz-Rivera et al. (51). The MIC for *E. coli* BW25113 was determined using broth microdilution (52), with bacterial growth monitored at OD_600_ using a microplate reader (Molecular Device, San Jose, USA).

### High-throughput screening of sensitive and resistant deletion mutants

The Keio collection was screened using the Singer RoToR HDA robotic system (Singer, Somerset, UK). Mutants were cultured on LB agar with reuterin (0×–2× MIC) and monitored using the Colony-live2 platform (11). Growth parameters (CONV, LTG, MGR, and SPG) were derived from Gompertz model fitting. Experimental reproducibility was assessed using the intraclass correlation coefficient analysis (53). Detailed protocols are provided in Supplemental methods.

### Bioinformatics analysis

Functional annotation and pathway analysis were performed using Clusters of Orthologous Groups (54), Gene Ontology (55), DAVID (56), and KEGG (57) databases. Protein-protein interaction networks were constructed using the STRING (16) database, and key hub proteins were identified by applying four topological algorithms: Degree, Maximal Clique Centrality, Betweenness, and Closeness (58) to evaluate node connectivity, centrality, and information flow across functional modules.

### Statistical analysis

**Fig 7:**
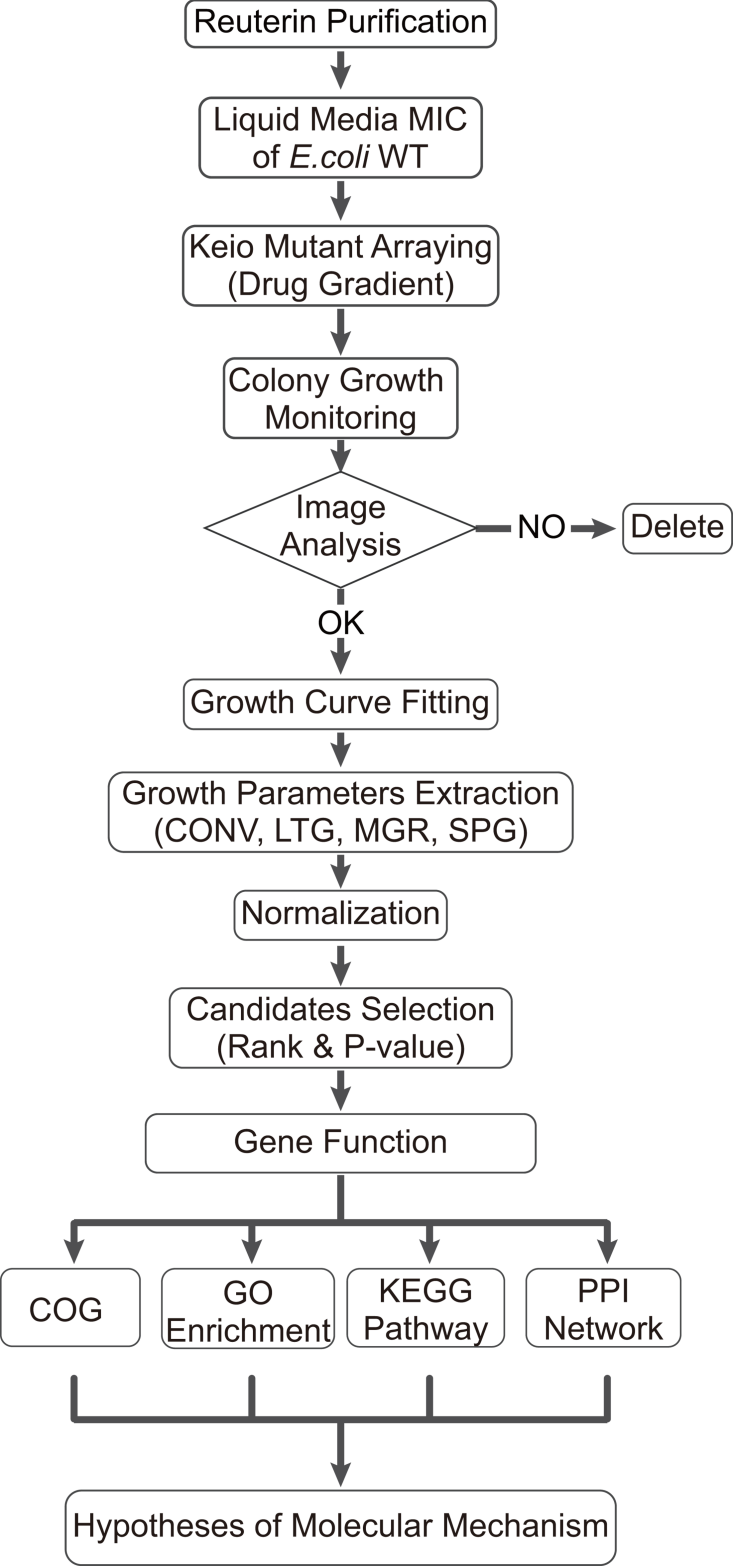
Workflow used to screen reuterin sensitivity. This workflow depicts high-throughput screening of the Keio collection to assess reuterin sensitivity and resistance. The process starts with determining the minimum inhibitory concentration of reuterin for K-12 BW25113. Mutants are then spiked onto reuterin-containing media, and colony growth is monitored using the Colony-live2 system. Growth parameters such as colony area, lag time of growth, maximum growth rate, and saturation point growth are then extracted. After normalization, mutants are ranked and selected according to sensitivity or resistance, followed by analysis of gene functions for hypothesis development.

Statistical analyses and data visualization were conducted using Python and R. Visualization of specific data and network analyses were performed by SRplot (59) and Cytoscape (60), followed by the construction of gene interaction networks and the exploration of biological functions. The workflow is shown in Fig. 7, and each of the thresholds is presented in the results.

## Data Availability

All data are available in the supplemental material.

## SUPPLEMENTAL MATERIAL

Fig. S1 mbio.01432-25-s0001.pdf MIC of reuterin for *E. coli* BW25113 in liquid culture.

Fig. S2 mbio.01432-25-s0002.pdf Growth curves of sensitive and resistant mutants undertreated with reuterin.

Fig. S3 mbio.01432-25-s0003.pdf KEGG pathway enrichment analysis.

Fig. S4 mbio.01432-25-s0004.pdf PPI network analysis based on degree value.

Supplemental methods mbio.01432-25-s0005.pdf Detailed methods.

Table S1 mbio.01432-25-s0006.xlsx High-throughput screening raw data.

Table S2 mbio.01432-25-s0007.xlsx Screened candidates.

Table S3 mbio.01432-25-s0008.xlsx Mapping of the candidates on the KEGG.

ASM does not own the copyrights to Supplemental Material that may be linked to, or accessed through, an article. The authors have granted ASM a non-exclusive, world-wide license to publish the Supplemental Material files. Please contact the corresponding author directly for reuse.
