# Supplementary material for: Systematic analysis of the antibacterial mechanisms of reuterin using the E. coli Keio collection
Source: mBio. 2025 Jul 3;16(8):e01432-25. doi: 10.1128/mbio.01432-25 (PMC12345186; doi:10.1128/mbio.01432-25)
Supplement: Fig. S1 — MIC of reuterin for E. coli BW25113 in liquid culture. [file mbio.01432-25-s0001.pdf]

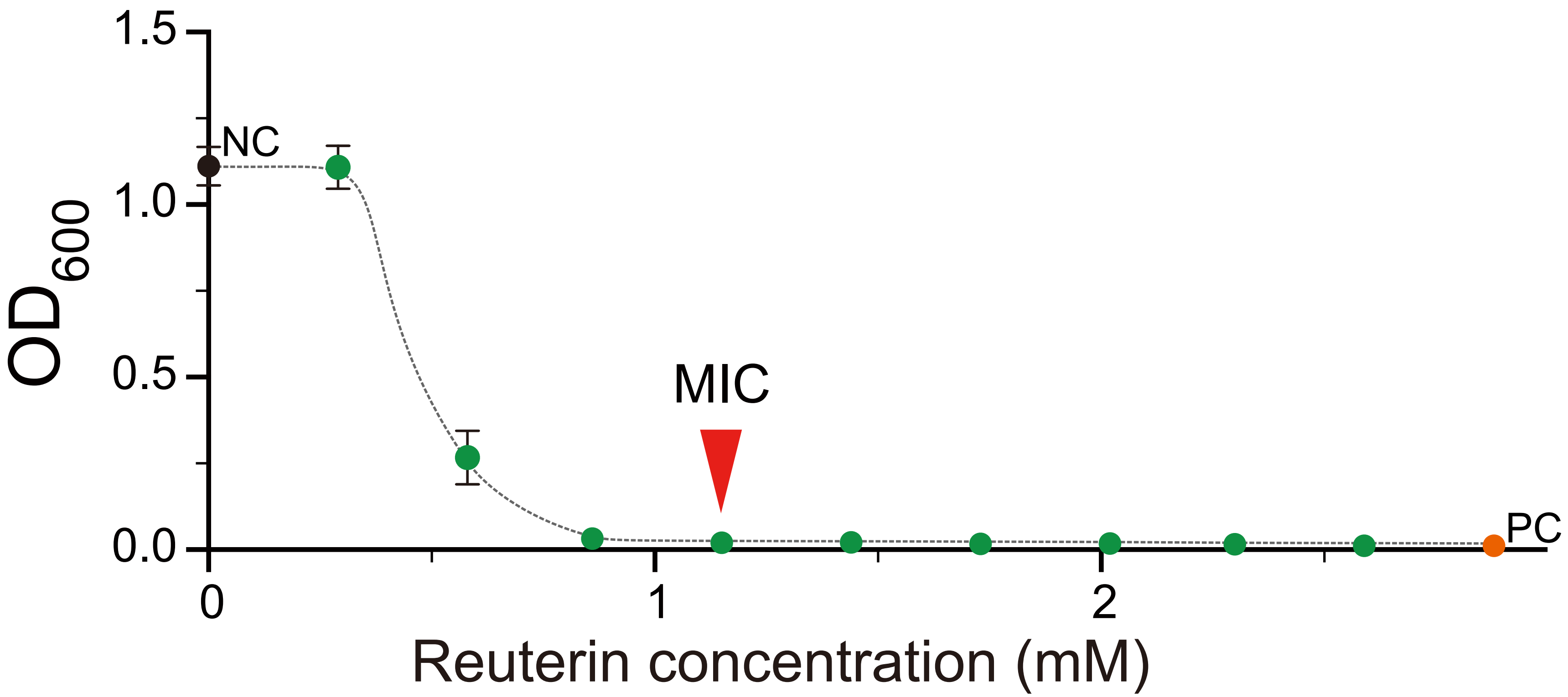

Figure S1. MIC of reuterin for *E. coli* BW25113 in liquid culture. The positive control (PC) was 30 μg/mL kanamycin, and the negative control (NC) was LB medium.
