# Supplementary material for: Systematic analysis of the antibacterial mechanisms of reuterin using the E. coli Keio collection
Source: mBio. 2025 Jul 3;16(8):e01432-25. doi: 10.1128/mbio.01432-25 (PMC12345186; doi:10.1128/mbio.01432-25)
Supplement: Fig. S2 — Growth curves of sensitive and resistant mutants undertreated with reuterin. [file mbio.01432-25-s0002.pdf]

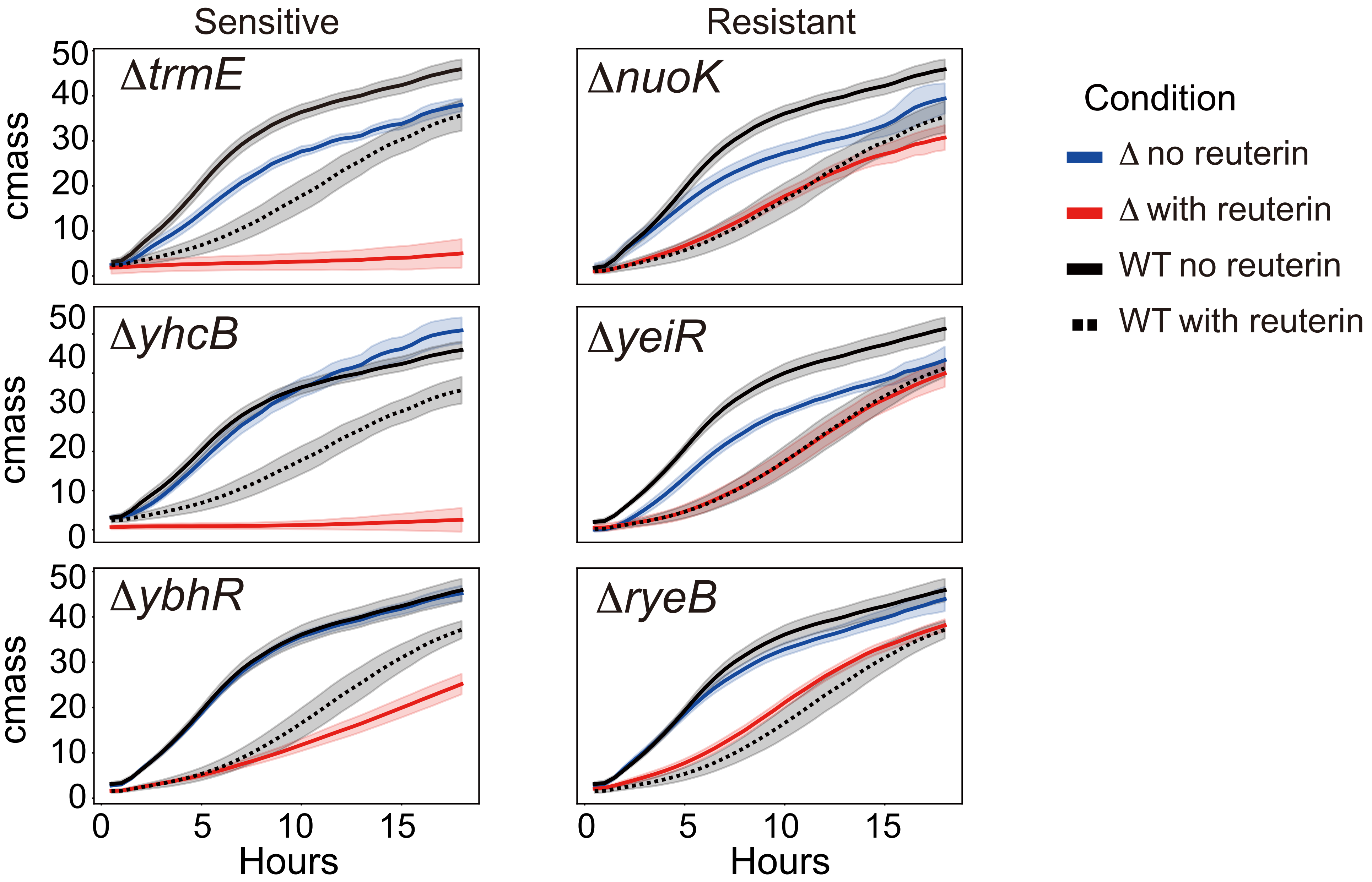

Figure S2. Growth curves of sensitive and resistant mutants under treated with reuterin (0.5 × MIC). Curve fitting was conducted for six representative mutants from the sensitive and resistant groups.
