# Supplementary material for: Systematic analysis of the antibacterial mechanisms of reuterin using the E. coli Keio collection
Source: mBio. 2025 Jul 3;16(8):e01432-25. doi: 10.1128/mbio.01432-25 (PMC12345186; doi:10.1128/mbio.01432-25)
Supplement: Fig. S3 — KEGG pathway enrichment analysis. [file mbio.01432-25-s0003.pdf]

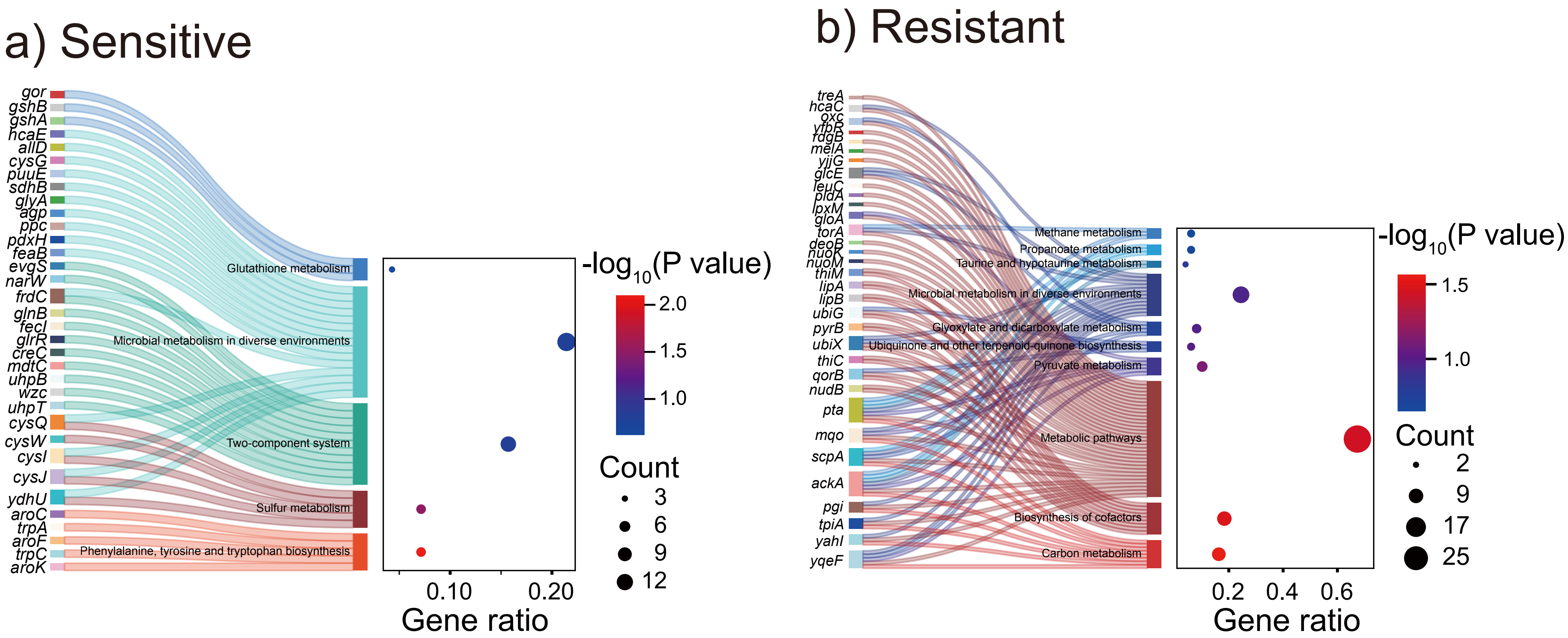

Figure S3. KEGG pathway enrichment analysis of genes from the sensitive and resistant groups. a) KEGG enrichment analysis of sensitive genes. The x-axis represents the gene ratio, the bubble size represents the number of genes in the pathway, and the bubble color represents the -log10 (P value). The Sankey diagram shows enriched pathways and their association with the genes. b) KEGG enrichment analysis of resistant genes. Similar to a), showing the distribution and association of resistant genes across enriched pathways.
