# Supplementary material for: Systematic analysis of the antibacterial mechanisms of reuterin using the E. coli Keio collection
Source: mBio. 2025 Jul 3;16(8):e01432-25. doi: 10.1128/mbio.01432-25 (PMC12345186; doi:10.1128/mbio.01432-25)
Supplement: Fig. S4 — PPI network analysis based on degree value. [file mbio.01432-25-s0004.pdf]

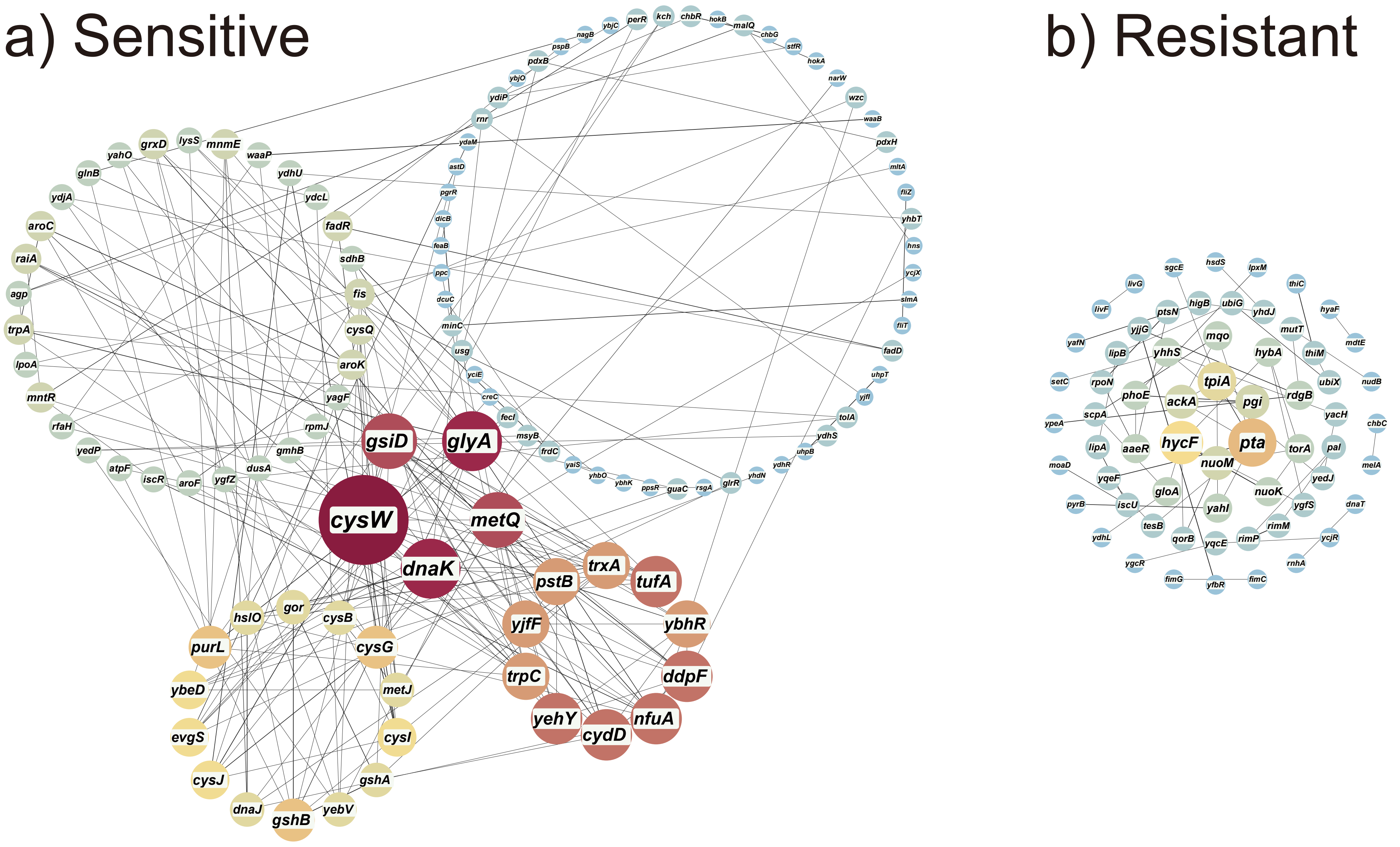

Figure S4. PPI network analysis based on Degree value. a) Sensitive group. b) Resistant group. The increase in node size and the gradient transition of node color (from blue to yellow to red) reflect an increase in Degree values.
