# Supplementary material for: Systematic analysis of the antibacterial mechanisms of reuterin using the E. coli Keio collection
Source: mBio. 2025 Jul 3;16(8):e01432-25. doi: 10.1128/mbio.01432-25 (PMC12345186; doi:10.1128/mbio.01432-25)
Supplement: Supplemental methods — Detailed methods. [file mbio.01432-25-s0005.pdf]

Supplementary methods

Preparation of reuterin

Reuterin was prepared according to Doleyres et al. (1) with modifications:

1. Culture conditions: *Limosilactobacillus reuteri* ATCC 55730 was cultured anaerobically (without shaking) in 1000 mL MRS broth (pH 6.0) supplemented with 20 mM glycerol at 37 ˚C for 16 h.
2. Cell harvesting: Cells were pelleted by centrifugation at 7600 × g at 4 ˚C for 10 min and washed twice with ice-cold 100 mM phosphate buffer (pH 7.0).
3. Reuterin synthesis: Washed cells were resuspended in 300 mL sterile glycerol solution (200 mM) and incubated at 37 ˚C for 2.5 h.
4. Supernatant processing: The culture supernatant was collected by centrifugation at 7600 × g at 4 ˚C for 10 min, sterilized through the 0.22 μm filter, and reuterin concentration was quantified as described by Ortiz-Rivera et al. (2).

MIC determination

The minimum inhibitory concentration (MIC) of reuterin for *E. coli* BW25113 was determined using broth microdilution (3):

1. Bacterial culture preparation: A glycerol stock of *E. coli* (stored at -80 ˚C) was inoculated into 5 mL of LB and grown aerobically overnight (16 –18 h) at 37 ˚C with shaking at 150 rpm in a thermostatic water bath shaker (THZ-82A; Changzhou Aohua Instrument, China).
2. Microdilution assay: Reuterin was serially diluted in LB medium at a 1:2 ratio. A volume of 99 μL of each reuterin-LB mixture was transferred into individual wells

of a 96-well plate. Subsequently, 1 μL of the *E. coli* overnight culture was inoculated into each well. The plate was incubated at 37 ˚C for 24 h with shaking at 600 rpm.

3. Growth measurement and MIC determination: The optical density at 600 nm (OD_{600}) was measured using a microplate reader (Molecular Devices, San Jose, USA). The MIC was defined as the lowest concentration of reuterin that completely inhibited visible bacterial growth.

High-Throughput screening

1. Strain transfer and seed preparation

1.1 Library handling: The Keio collection (384-well glycerol stocks stored at -80 ˚C) was replicated onto LB agar plates supplemented with 30 μg/mL kanamycin using a Singer RoToR® HDA robotic system (Singer Instrument Inc., Somerset, UK) equipped with long-pin pads. Specifically, four glycerol stock plates were stamped onto a single LB agar plate using 384-pin pads, generating high-density arrays of 1,536 colonies per plate.

1.2 Seed plate preparation: The stamped plates were incubated at 37 ˚C for overnight to obtain 1,536-colony seed plates.

2. Reuterin treatment and growth monitoring

2.1 Drug exposure: Seed colonies were stamped onto LB agar plates containing 30 μg/mL kanamycin supplemented with 0 ×, 0.5 ×, 1 ×, and 2 × the MIC of reuterin using the 1,536 short-pin pads.

2.2 Image acquisition: Colony growth was monitored every 30 min for 18 h using the

Colony-live2 platform (an updated version of the original Colony-live system; manuscript in preparation).

3. Data analysis and reproducibility

3.1 Growth parameter extraction: Colony area (CONV) and mass (Mass) were quantified as previously reported (4). Gompertz model fitting was applied to calculate Lag Time of Growth (LTG), Maximum Growth Rate (MGR), and Saturation Point Growth (SPG) by using the continuous-time absorbance of the central 17 pixels for each colony (cmass).

3.2 Reproducibility assessment: Intraclass correlation coefficient (ICC) (5) was calculated via one-way ANOVA of four independent replicates in R.

3.3 Mutant classification: Sensitive/resistant mutants were identified using two methods:

1) Threshold-based selection: $\pm 2\sigma$ from the mean fold change ratio (presence/absence of reuterin) in MGR.

2) Statistical significance: Paired t-test ($P < 0.01$, fold change > 1 or < 1).

References of Supplementary Information

1. Doleyres Y, Beck P, Vollenweider S, Lacroix C. 2005. Production of 3-hydroxypropionaldehyde using a two-step process with *Lactobacillus reuteri.* Appl Microbiol Biotechnol 68:467-74.
2. Ortiz-Rivera Y, Sánchez-Vega R, Gutiérrez-Méndez N, León-Félix J, Acosta-Muñiz C, Sepulveda DR. 2017. Production of reuterin in a fermented milk product by *Lactobacillus reuteri*: Inhibition of pathogens, spoilage microorganisms, and lactic acid bacteria. J Dairy Sci 100:4258-4268.
3. Andrews JM. 2001. Determination of minimum inhibitory concentrations. J Antimicrob Chemother 48 Suppl 1:5-16.
4. Takeuchi R, Tamura T, Nakayashiki T, Tanaka Y, Muto A, Wanner BL, Mori H. 2014. Colony-

live--a high-throughput method for measuring microbial colony growth kinetics--reveals diverse growth effects of gene knockouts in *Escherichia coli*. BMC Microbiol 14:171.

5. Shrout PE, Fleiss JL. 1979. Intraclass correlations: uses in assessing rater reliability. Psychol Bull 86:420-8.
